# Metronidazole-Associated Encephalopathy

**DOI:** 10.4269/ajtmh.17-0574

**Published:** 2018-02

**Authors:** Daisuke Taniyama, Taketomo Maruki

**Affiliations:** Department of General Internal Medicine, Tokyo Saiseikai Central Hospital, Tokyo, Japan

**Figure 1.**
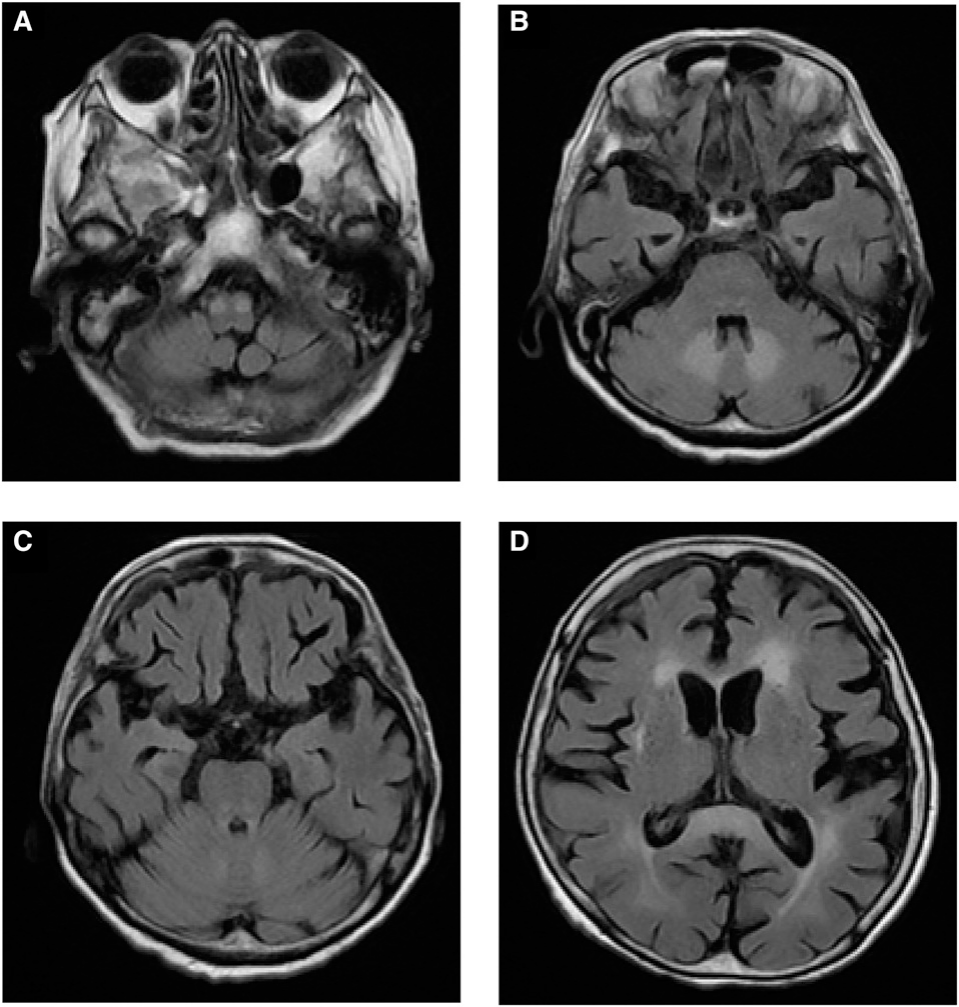
Fluid-attenuated inversion recovery magnetic resonance imaging after the start of metronidazole treatment. The scans after the start of metronidazole treatment reveal bilaterally symmetrical high signals in the (**A**) bulb olivary nucleus, (**B**) cerebellar dentate nucleus, (**C**) pons, and (**D**) splenium of the corpus callosum.

**Figure 2.**
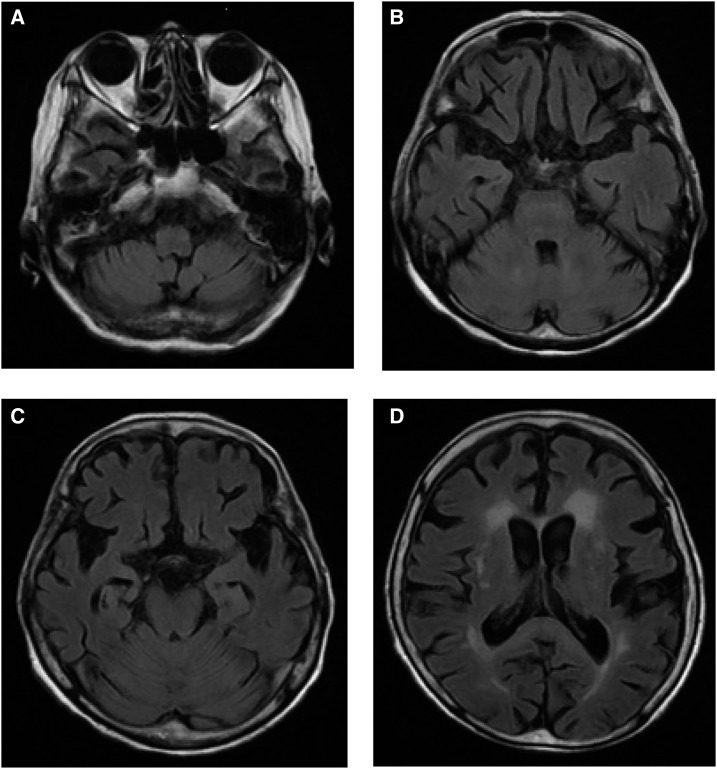
Fluid-attenuated inversion recovery magnetic resonance imaging after stopping metronidazole treatment. The scans 3 weeks after stopping the metronidazole treatment reveal resolution of bilaterally symmetrical high signals in the (**A**) bulb olivary nucleus, (**B**) cerebellar dentate nucleus, (**C**) pons, and (**D**) splenium of the corpus callosum.

An 89-year-old woman was admitted to our hospital with abscesses in her brain and liver due to *Fusobacterium* spp. She was administered metronidazole (total dose of 73.5 g) intravenously. Two months later, a fluid-attenuated inversion recovery magnetic resonance imaging (MRI) scan for evaluation of the brain abscess revealed bilateral, symmetrical high signals in the bulb olivary nucleus, cerebellar dentate nucleus, pons, and splenium of the corpus callosum, consistent with metronidazole-associated encephalopathy (MAE) (Figure 1A–D). Around the same time, she developed ataxia but other neurologic assessments were difficult because she was on bed rest. When the control of brain abscess was good, metronidazole treatment was stopped, which resulted in resolution of the MRI findings 3 weeks later (Figure 2A–D). Prolonged administration of metronidazole has been shown to cause toxicity,^1^ although differences in the effects of oral versus intravenous administration are undetermined.^2^ Since the recent entry of an intravenous form of metronidazole in the Japanese market, its use in the clinic has been gaining renewed interest, leading to an increase in the reports of MAE.^3^ Because long-term metronidazole therapy is often chosen to treat brain abscesses due to anaerobic bacteria, caution should be practiced owing to possible development of MAE.
